# Efficacy of Polyphenylene Carboxymethylene (PPCM) Gel at Protecting Type I Interferon Receptors Knockout Mice from Intravaginal Ebola Virus Challenge

**DOI:** 10.3390/v16111693

**Published:** 2024-10-30

**Authors:** Olivier Escaffre, Terry L. Juelich, Jennifer K. Smith, Lihong Zhang, Madison Pearson, Nigel Bourne, Alexander N. Freiberg

**Affiliations:** 1 Department of Pathology, University of Texas Medical Branch, Galveston, TX 77555, USA; 2 Institute for Human Infections & Immunity and Sealy & Smith Foundation, University of Texas Medical Branch, Galveston, TX 77555, USA; 3 Institute for Translational Sciences, University of Texas Medical Branch, Galveston, TX 77555, USA; 4 Department of Microbiology and Immunology, University of Texas Medical Branch, Galveston, TX 77555, USA; 5 Department of Pediatrics, University of Texas Medical Branch, Galveston, TX 77555, USA; 6 Sealy Institute for Vaccine Sciences, University of Texas Medical Branch, Galveston, TX 77555, USA; 7 Center for Biodefense and Emerging Infectious Diseases, University of Texas Medical Branch, Galveston, TX 77555, USA

**Keywords:** Ebola virus, microbicide, PPCM, sexual transmission, intravaginal infection, IFNAR^−/−^ mice

## Abstract

Ebola virus (EBOV) is one of three filovirus members of the *Orthoebolavirus* genus that can cause severe Ebola disease (EBOD) in humans. Transmission predominantly occurs from spillover events from wildlife but has also happened between humans with infected bodily fluids. Specifically, the sexual route through infectious male survivors could be the origin of flare up events leading to the deaths of multiple women. More studies are needed to comprehend this route of infection which has recently received more focus. The use of microbicides prior to intercourse is of interest if neither of the Ebola vaccines are an option. These experimental products have been used against sexually transmitted diseases, and recently polyphenylene carboxymethylene (PPCM) showed efficacy against EBOV in vitro. Shortly after, the first animal model of EBOV sexual transmission was established using type I interferon receptors (IFNAR^−/−^) knockout female mice in which mortality endpoint could be achieved. Here, we investigated PPCM efficacy against a mouse-adapted (ma)EBOV isolate in IFNAR^−/−^ mice and demonstrated that 4% PPCM gel caused a 20% reduction in mortality in two distinct groups compared to control groups when inoculated prior to virus challenge. Among animals that succumbed to disease despite PPCM treatment, we report an increase in median survival time as well as a less infectious virus, and fewer virus positive vaginal swabs compared to those from vehicle-treated animals, altogether indicating the beneficial effect of using PPCM prior to exposure. A post-study analysis of the different gel formulations tested indicated that buffering the gels would have prevented an increase in acidity seen only in vehicles, suggesting that PPCM antiviral efficacy against EBOV was suboptimal in our experimental set-up. These results are encouraging and warrant further studies using optimized stable formulations with the goal of providing additional safe protective countermeasures from sexual transmission of EBOV in humans.

## 1. Introduction

Ebola disease (EBOD) in humans is caused by zoonotic RNA filoviruses of the *Orthoebolavirus* genus, including the *Orthoebolavirus sudanense* (SUDV), *zairense* (EBOV), and *bundibugyoense* (BDBV) species [1,2,3]. Although more than 28,000 cases of EBOD were reported between 2014–2016, outbreaks are generally smaller in size with high mortality rates. The last Ebola outbreak was reported in 2023 in Uganda from SUDV infection and caused Sudan virus disease (SVD) in 164 cases with 34% mortality [4]. Historically, about 25% of EBOD outbreaks between 1976 and 2022 have resulted from a nosocomial infection [5], but EBOV was also suspected to be sexually transmitted to women and caused flare up events during the largest EBOV outbreak in history in West Africa [4]. Despite its initial discovery back in 1976 [6], little is still known regarding how much this latter route of transmission effectively contributes to EVD spread. Although considered as a rare disease, prophylactic methods are much needed in view of the high mortality rate, how long the virus genome can remain in the reproductive system of male survivors [7,8,9,10,11,12,13,14,15], as well as the cost of an EVD case not surviving [16]. There are currently two licensed EBOV vaccines (Ervebo^®^ and Zabdeno^®^/Mvabea^®^), which are primarily available to high-risk groups. Furthermore, acceptance of healthcare workers or patients have sometimes made their use challenging [17,18,19], although efforts have been made to repurpose doses for preventive vaccination [20].

Microbicides are meant to be delivered into the vagina and carry active ingredients that are broad-spectrum, virus- or bacteria-specific [21,22,23,24,25,26,27,28]. In the context of EBOV sexual transmission, PPCM could be effective pre-exposure prophylaxis as an alternative method to no protection at all or abstinence and could be used in combination with condoms or other antiviral agents. However, none have currently received regulatory approval for EBOV due to the lack of scientific data. Polyphenylene carboxymethylene (PPCM) is a polymer licensed to Yaso Therapeutics and has previously experimentally shown anti-EBOV activity in vitro [29,30], but no corresponding data are currently available in animal models. Interestingly, type I interferon receptors (IFNAR^−/−^) knockout mice were recently shown to exhibit significantly more clinical signs of disease than BALB/c mice [31,32] following intravaginal EBOV challenge and thus constitute a more suitable model for therapeutic testing.

Here, we report the first study evaluating the efficacy of PPCM microbicide gel against EBOV in the context of a sexually transmitted disease in IFNAR^−/−^ knockout female mice.

## 2. Materials and Methods

Ethics: The study was approved by the Institutional Animal Care and Use Committee at UTMB (protocol 2104028, approved in 2021) and conducted in accredited facilities (Association for Assessment and Accreditation of Laboratory Animal Care International, AAALAC), as previously described [31].

Virus and cells: Infectious work was conducted at biosafety level 4 at UTMB. Details on mouse-adapted Ebola virus (maEBOV) origin, stock preparation, and titration were previously described [31]. Briefly, maEBOV was originally obtained from serial passages in mice and then Vero E6 cells (ATCC, CRL1586) before making a working stock by sucrose cushion purification. Virus stock and infectious samples from this study were titrated by a conventional plaque assay technique in Vero E6 cells and titers expressed as log10 in pfu/mL.

Study design: 50 18–23-week-old female mice lacking type I interferon receptors (IFNAR^−/−^, 129Sv/Black Swiss background) were obtained from a colony at UTMB (originally provided by Dr. Michael Diamond, Washington University School of Medicine, St. Louis, MO, USA) and divided into 5 groups (n = 10). Consistent with previous studies [31,32], all subjects received 3 mg of progesterone acetate by subcutaneous injection 6 days prior to virus challenge. On study day 0 (SD0), mice from the 5 groups were anesthetized for about 35 min by injectables (60–75 mg/kg of ketamine and 6–7.5 mg/kg of xylazine) given intraperitoneally. This method was previously used to perform intravaginal infections [33] and was necessary in the present study to perform a one-time inoculation of 25 μL per subject of vehicle or microbicide gel into the vaginal vault of animals from 4 groups followed by 25 min of incubation with animals in the supine position. Note that effective delivery of drug and vehicle was ensured by using a positive displacement pipette with proper capillary/piston. Specifically, 2 groups received the vehicle gels made of either 0.15 or 0.06 mg/mL xanthan gum (high and low viscosity, respectively). This gum is commonly used in the food, cosmetic, and pharmaceutical industries as a thickener. Two other groups received the corresponding high- and low-viscosity microbicide gels containing 4% PPCM as the active ingredient. Gels were provided by Yaso Therapeutics and made under CGMP by Dow Development Laboratories. A dose of 10^4^ particle-forming units (PFU) per subject of maEBOV (10 μL virus in PBS) was then administered intravaginally to animals of these 4 groups. The virus was allowed to incubate for a minimum of 5 min in anesthetized animals placed in the supine position prior to returning to their cages. One control group remained non-treated and non-infected throughout the study. Three animals from that group were euthanized on the first day of moribundity seen in any of the 4 infected groups. Three more control subjects were also euthanized 2 days later. The study lasted for 21 days post-challenge, during which weight loss was monitored and animals scored for signs of disease (lethargy, ruffled fur, hunched posture, orbital tightening). Euthanasia was required if a subject displayed a moribund appearance, ≥20% weight loss, reluctance to move when stimulated, paralysis, or inability to access food and water. Vaginal swabs were performed every 2 days or when the euthanasia criteria were reached, similar to our previous study [31].

Neutralization assay: Serum neutralization assays were performed on heat-inactivated samples from naïve and maEBOV-infected mice. Positive controls of EBOV neutralization were obtained from our previous serial dose study using BALB/c mice [31]. Briefly, samples were then serially diluted (starting dilution 1/10) and incubated with the virus (80 plaques per 100 μL) for 1 h at 37 °C, 5% CO_2_. Mixtures were then incubated with confluent Vero E6 cells in 12-well plates for an additional hour at 37 °C, 5% CO_2_ prior to adding tragacanth/MEM 2% FBS overlay, as previously described [31]. The plaque count was determined 13 days post-infection. The neutralization titer (PRNT_50_) corresponds to the reciprocal dilution at which the virus plaque count is reduced by half when compared to the viral control. Samples with no plaque reduction at 1/10 dilution were considered seronegative (PRNT_50_ = 0).

maEBOV RT-qPCR: Tissues for which the virus titer remained under limit of detection (LOD) by the plaque assay technique (LOD of 10^1.82^ PFU/mL) were also processed for quantitative reverse transcription polymerase chain reaction with 40 cycles targeting the virus’s glycoprotein gene. Specifically, the other half of each tissue was placed in TRIzol reagent (Thermo Fisher Scientific, California, USA) and processed with a TissueLyser (Qiagen, Hilden, Germany). The total RNA from the clarified tissue homogenates was extracted and purified using Direct-zol RNA miniprep (Zymo Research, Irvine, USA), following the manufacturer’s recommendations. Ten-fold serially diluted RNA standard curves were prepared from the virus stock using the same extraction method and were plotted to their known equivalent PFU per reaction, ranging from 2.9 × 10^5^ to 2.9 × 10^−1^, to determine sample concentration. The equivalent concentration of the virus in tissues was then adjusted per gram. The sequences for the probe, and forward and reverse primers were CATGTGCCGCCCCATCGCTGC, TTTTCAATCCTCAACCGTAAGGC, and CAGTCCGGTCCCAGAATGTG, respectively.

Blood chemistry: Blood samples were processed in EDTA and serum separator (SST) collection tubes to perform hematology and biochemistry analysis using an HEMAVET hematology analyzer (Drew Scientific, Plantation, USA) and mouse comprehensive diagnostic profile rotors on a VetScan Chemistry Analyzer (Abaxis, Union City, USA), respectively.

Bio-plex assay: Sera from SST tubes were virus-inactivated by gamma irradiation (5Mrad dose) on dry ice, as previously described [30]. Samples were run on a Bio-Plex Pro Mouse Cytokine Grp 1 panel 23-plex (Bio-Rad, Hercules, USA) using a Bio-Plex 200 system. Inflammatory markers included IL-1α, IL-1β, IL-2, IL-3, IL-4, IL-5, IL-6, IL-9, IL-10, IL-12 (p40), IL-12 (p70), IL-13, IL-17, Eotaxin, G-CSF, GM-CSF, IFN-γ, KC, MCP-1, MIP-1α, MIP-1β, RANTES, and TNF-α.

Statistical analysis: The Mantel–Cox test was used to compare survival curves. Fisher’s exact test was used to compare the number of virus-positive swabs on a given day. The Koopman asymptotic score and the Newcombe–Wilson score method with continuity correction were used to compute confidence intervals (CI) for the relative risk and number needed to treat (NNT), respectively. An unpaired *t*-test or one-way ANOVA followed by Tukey’s multiple comparisons test was used to compare infectious virus titers from swabs and tissues as well as to compare hematology, biochemistry, and inflammatory parameters between groups (* *p* < 0.05, ** *p* < 0.01, *** *p* < 0.001).

## 3. Results

**Figure 1:**
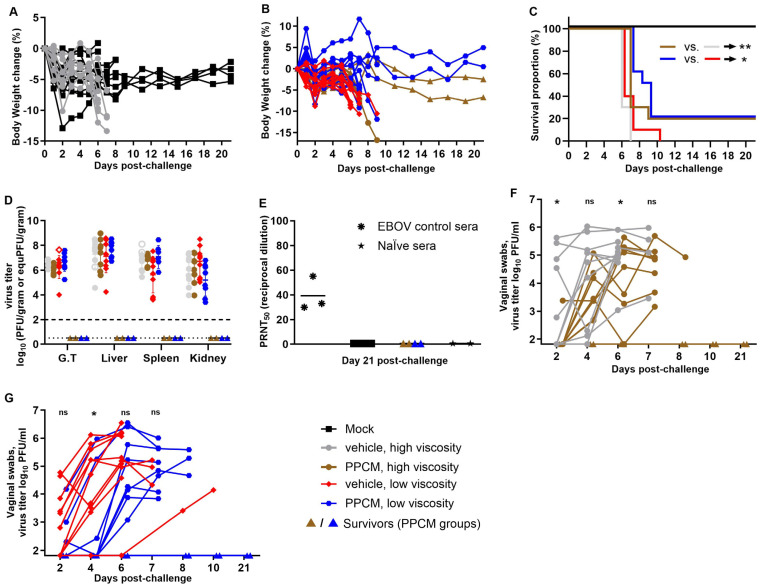
Intravaginal maEBOV challenge in progesterone-primed IFNAR^−/−^ mice and PPCM efficacy at preventing infection. Four groups of mice (n = 10/group) received a topical gel intravaginally (vehicle or PPCM) and were then challenged with a 10^4^ pfu dose and monitored for 21 days for weight loss (**A**,**B**), and mortality (**C**) as well as virus dissemination when moribund (**D**). Open symbols (**D**) are values obtained by RT-qPCR and substitute the value of undetected virus from titrating by plaque assay the other half of the corresponding tissue. Seroconversion was evaluated at day 21 post-challenge and PRNT_50_ was calculated when possible (**E**). Shedding of virus was assessed longitudinally using vaginal swabs starting on day 2 (**F**,**G**). Note that replicates in (**F**,**G**) are aligned for better tracking of individual values over time and some symbols may overlap in the early time points (n = 10 in all groups at days 2 and 4). Depending on the assay, the limit of detection was 10^2^ PFU/gram (**D**, thick horizontal dotted line) or 10^1.82^ PFU/mL (**F**,**G**) for titration by plaque assay, or 10^0.5^ equivalent (equ) PFU/gram (**D**, thin horizontal dotted line) by RT-qPCR analysis with the virus stock used as standard. Subjects are represented by individual symbols in (**A**,**B**,**D**,**E**), and may overlap in (**F**,**G**). The Mantel–Cox test was applied to survival data. The *t*-test was applied to titration data from swabs. Asterisks (* or **) indicate statistical differences of *p* < 0.05 or 0.01, respectively, in the survival data (**C**) or average virus titers from swabs (**F**,**G**) between groups. (ns) for non-significant. Abbreviation: Genital tract (G.T.).

Mice from four groups (each n = 10) received an effective dose of 9.32 × 10^3^ PFU/subject (target dose of 10^4^) by intravaginal route following a 25-min incubation period with either 4% PPCM gels or corresponding vehicles of low or high viscosity. Two viscosities were tested, as surface coverage of the vaginal wall coated with drug might be impacted. One additional group served as a negative control and underwent the anesthesia process without further manipulations. Weight loss was generally minimal during the first two days in all groups (<5% change) except for one subject each in the negative control (−12.9%), vehicle (high viscosity) (−10.1%), and PPCM (low viscosity) (−8.3%) groups (Figure 1A,B), which likely resulted from the anesthesia. Body weights then remained stable or slightly increased for all animals until day 5 post-infection (dpi) but began to decrease thereafter for the subjects that eventually succumbed to disease or met euthanasia criteria. Specifically, lethality was seen in 100% of vehicle and 80% of PPCM groups between days 6 and 10. Indeed, four subjects (two per group) from the PPCM-treated groups remained healthy until the end of the study, which is a small but significant difference in mortality rate between matching groups (*p* < 0.05 or 0.01) (Figure 1C). The manifestation of the disease was comparable between groups in terms of severity and type of clinical signs including lethargy, ruffled fur, hunched posture, orbital tightening, and moribund appearance. No vaginal bleeding was recorded, as opposed to our previous observations in BALB/c mice [31]. A comparable amount of infectious virus was found in the genital tract (G.T.), liver, spleen, and kidney from animals that succumbed to infection or met euthanasia criteria, irrespective of the group (Figure 1D). Note that four tissues (out of 144), whose infectious titer was below the limit of detection, were at least positive for virus genome (open symbols, Figure 1D). However, no virus titer or genome could be retrieved from survivor tissues, suggesting that PPCM prevented complete infection in some subjects, which was consistent with their absence of seroconversion to challenge (Figure 1E). Note that difference in gel viscosity did not affect outcome.

However, median survival time for PPCM-treated groups was longer by 1 to 2.5 days than that of vehicle-treated groups. Specifically, the median survival was 6 days in both the vehicle-treated group and 7 or 8.5 days in the high- and low-viscosity PPCM-treated groups (Figure 1C), respectively, suggesting that PPCM could at a minimum delay the infection, which was best observed using the lowest viscosity. In line with this, the average virus titer recovered from vaginal swabs was higher at days 2, 4, or 6 post-challenge (*p* < 0.05) in the vehicle- compared to the PPCM-treated group using the high- or low-viscosity formula (Figure 1F,G). When merging data, regardless of viscosity (n = 20 for both vehicle- and PPCM-treated groups), the number of EBOV positive vaginal swabs (≥10^1.82^ PFU/mL) at day 2 was not different, although approaching significance (*p* = 0.0536), but it was higher in the vehicle-treated group (*p* = 0.019) by day 4 (Supplementary Table S1), supporting the differences in median survival data and thus the role of PPCM in delaying disease. In fact, a mouse treated with PPCM was only 63% as likely to have an EBOV-positive vaginal swab as one treated with vehicle (relative risk), indicating a beneficial effect of using PPCM. Further data analyses also showed that number of mice that needed to be treated (NNT) with PPCM to prevent detection of a positive EBOV swab in one additional mouse was 2.9 (Supplementary Table S1).

Examination of the pH of leftover gels indicated a significant drop by one unit or more only in vehicle formulations between the time of manufacturing and the end of the animal study (from 5.9 to 4.45 or from 6.5 to 5.5), suggesting a tenfold increase in acidity that the PPCM active ingredient prevented in PPCM gels by acting as a buffer. Changes in PPCM ionization to maintain an otherwise decreasing pH likely reduced PPCM’s overall charge and potentially lowered its efficacy at binding to EBOV glycoprotein or masking EBOV cell receptors [29].

**Figure 2:**
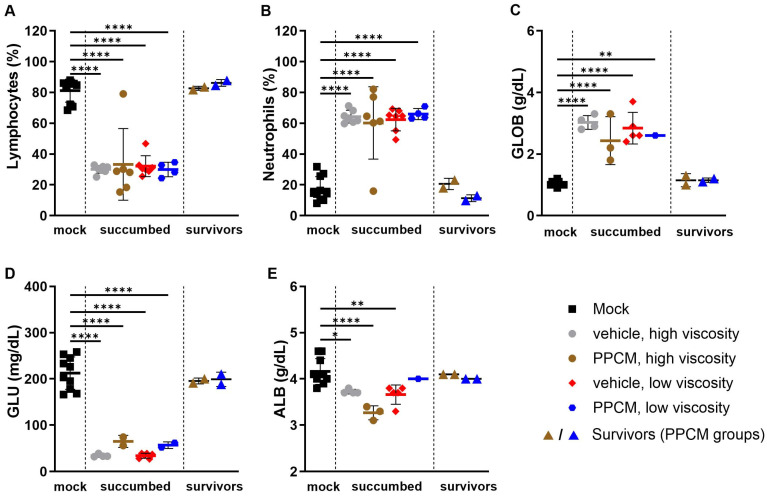
Hematology and biochemical profile of blood from IFNAR^−/−^ mice following intravaginal maEBOV challenge. Bar graphs represent lymphocyte (**A**) and neutrophil (**B**) populations from whole blood analyses as well as levels of globulin (**C**), glucose (**D**), and albumin (**E**) in sera at a predetermined time or when the subject became moribund. Subjects are represented by individual symbols and error bars show standard deviations. Note that not all parameters could be determined from a sample due to low volume or hemolysis. A one-way ANOVA, followed by Tukey’s multiple comparisons test, was used. Asterisks (*, **, ****) indicate statistical differences of *p* < 0.05, 0.01, or 0.0001, respectively, for a given parameter between groups.

**Figure 3:**
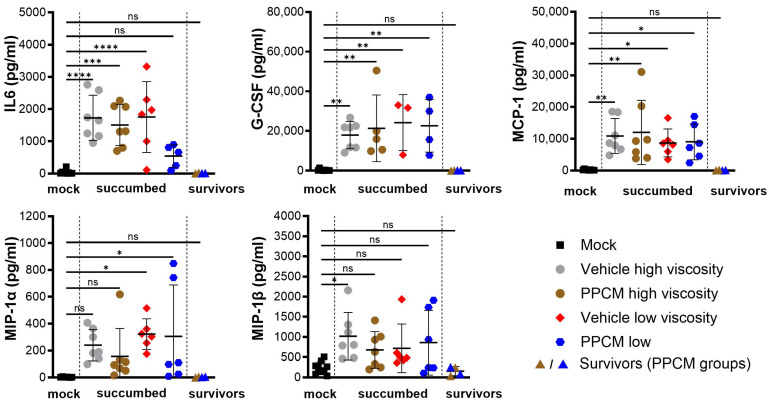
Circulating inflammatory markers resulting from intravaginal maEBOV challenge in progesterone-primed IFNAR^−/−^ mice. Serum samples were collected at a predetermined time or when the subject became moribund and were then assessed by multiplex immunoassays. Subjects are represented by individual symbols, and error bars show standard deviations. Note that not all samples were available due to low blood volumes collected from moribund animals. A one-way ANOVA, followed by Tukey’s multiple comparisons test, was used. Asterisks (*, **, ***, ****) indicate statistical differences of *p* < 0.05, 0.01, 0.001, or 0.0001, respectively, for a given analyte between groups. (ns) for non-significant.

Consistent with Ebola virus disease (EVD) in mice, the majority of subjects that succumbed to disease or required euthanasia, regardless of the group, presented with lymphopenia (*p* < 0.0001), neutrophilia (*p* < 0.0001) (Figure 2A,B), as well as kidney and liver damage as determined by increased globulin (*p* < 0.01 to 0.0001) and low levels of glucose and albumin (*p* < 0.05 to 0.0001) in the blood (Figure 2C–E). Interestingly, corresponding data from EBOV survivors were comparable to those of mock samples collected throughout the study, which is in line with an absence of seroneutralization and virus in all survivor specimens. Increased secretion of virus-induced inflammatory molecules including IL-6, G-CSF, MCP-1, and MIP-1α/β was found in subjects that succumbed to disease compared to those from mock and survivor groups (non-significant to *p* < 0.0001) (Figure 3). This is consistent with an increase in granulocyte count and activation of other immune cells including macrophages, monocytes, and dendritic cells in the acute phase of infection. In addition, this provides further evidence of the potential prophylactic effect of PPCM against maEBOV, as values in mock and survivors were comparable.

## 4. Discussion

Male-to-female Ebola virus sexual transmission has been suspected in several outbreaks that resulted in fatalities [9,14,34,35,36], and this route of transmission for filoviruses is still vastly understudied. Vaccination, abstinence, and safe sexual practices such as using condoms, being mutually monogamous, and getting tested are effective ways to prevent transmission, but they are not commonly used and accepted [34,37,38,39]. Thus, there is still a need to develop new EBOV countermeasures, especially for women. We previously demonstrated PPCM drug efficacy against EBOV in vitro [29,30]. Here, we present the first study evaluating the efficacy of PPCM in a gel formulation against an assisted intravaginal maEBOV challenge in IFNAR^−/−^ mice.

Susceptibility to intravaginal maEBOV challenge was consistent with what was previously described using the same model in terms of time to death [32]. The uniform lethality seen in the present study in two distinct vehicle groups using 10^4^ PFU was necessary to assess the potential beneficial use of PPCM, and since 80% mortality was previously achieved using a lower dose [32], this suggests a dose-virulence correlation, as previously noted with other mouse models and routes of infection [40,41]. We also confirmed changes in lymphocyte and granulocyte counts, basic biochemical parameters of liver and spleen condition, as well as secretions of inflammatory mediators in subjects that succumbed to an intravaginal challenge, which is in line with previous studies in mice [41,42] and also relevant to EVD in humans [43].

Previously, we showed quasi-uniform seroconversion (25/26, 96%) of BALB/c mice following a 10^4^ PFU or lower intravaginal maEBOV challenge; however, the absence of seroconversion could still be paired with positive vaginal swabs for infectious virus at days 6 and 8 [31]. Interestingly, no seroconversion was observed in IFNAR^−/−^ survivors (n = 2, 0%) from a 10^3^ FFU dose of maEBOV [32], and the authors suspected no infection, suggesting that the minimal dose for infection and seroconversion is mouse-strain specific. Here, we did not observe any seroconversion in IFNAR^−/−^ survivors following a higher challenge dose either. However, we believe this is likely the result of PPCM effectively preventing infection, rather than not adequately challenging animals, whether it is dose- or method-related, for these four subjects kept in four distinct cages throughout the study. This is supported by the fact that the four survivors across 4 groups of 10 belonged to PPCM groups. Furthermore, a delay in time to death as well as lower virus titers from swabs or fewer positive swabs were reported in PPCM- compared to vehicle-treated groups.

ZMapp immunotherapy lowered the relative risk of death by 40% during the 2014 West Africa outbreak [44]. The mortality rate was even more significantly attenuated when using mAb114 (now Ebanga^TM^) and REGN-EB3 (now Inmazeb^TM^) compared to ZMapp during another trial in 2019 [45]. The PPCM gels tested in the present mouse study comparatively offered less protection and are not EBOV-specific [23,24,25,26,27], but efficacy of this polymer might be dependent on formulation. In vitro, a 4% solution in PBS could prevent EBOV replication and detection in a human model of the vaginal epithelium following a high infective dose (multiplicity of infection 5) [30]. A similar robust antiviral effect was also observed using a lower concentration diluted in cell culture medium in other human models [29]. Here, only 20% of subjects survived the challenge when PPCM gel was made in a non-buffered formulation. While it is unclear how tightly in vitro and in vivo data should correlate, further testing of PPCM is warranted considering its low cost and excellent safety profile in rabbits, rats, and mice [25,27]. This includes buffering gels to prevent a pH drop and change in PPCM ionization state, which in turn could optimize its bioavailability as a microbicide.

## Data Availability

The original contributions presented in this study are included in the article/Supplementary Material. Further inquiries can be directed to the corresponding authors.

## Supplementary Materials

The following supporting information can be downloaded at: https://www.mdpi.com/article/10.3390/v16111693/s1, Table S1: Vaginal swabs results following maEBOV intravaginal challenge.
