# Supplementary material for: Efficacy of Polyphenylene Carboxymethylene (PPCM) Gel at Protecting Type I Interferon Receptors Knockout Mice from Intravaginal Ebola Virus Challenge
Source: Viruses. 2024 Oct 30;16(11):1693. doi: 10.3390/v16111693 (PMC11598907; doi:10.3390/v16111693)
Supplement: Supplementary file 1 [file viruses-16-01693-s001.zip › viruses-3243104-supplementary.pdf]

Table S1. Vaginal swabs results following maEBOV intravaginal challenge.

Treatment \ Testing result	Vaginal swabs, Day 2		Vaginal swabs, Day 4		Vaginal swabs, Day 6	
	Under LOD	Detectable	Under LOD	Detectable	Under LOD	Detectable
Vehicle group (high and low viscosity)	8	12	1	19	1	19
PPCM group (high and low viscosity)	15	5	8	12	4	16
Fisher's exact test						
Statistically significant?	No (p = 0.0536)		Yes (p = 0.0197)		No (p = 0.3416)	
Relative risk	N/A		0.63 (95% CI 0.40 to 0.87)		N/A	
Number Needed to Treat	N/A		2.9 (95% CI 1.5 to 9.1)		N/A	

Abbreviations: limit of detection (LOD); confidence interval (CI); non applicable (NA).
